# Pain Management After Cesarean Delivery Among Women with Opioid Use Disorder: Results from a Retrospective Pregnancy Cohort in a Rural Region of the Midwest

**DOI:** 10.1089/whr.2022.0108

**Published:** 2023-04-13

**Authors:** Julia Riddle, Julie A. Botsford, Samantha Dean, Carol Coffman, Chelsea A. Robinson, Jean M. Kerver

**Affiliations:** ^1^ Grand Traverse Women's Clinic, Munson Healthcare, Traverse City, Michigan, USA.; ^2^ Pharmacy, Munson Healthcare, Traverse City, Michigan, USA.; ^3^ Clinical and Business Intelligence, Munson Healthcare, Traverse City, Michigan, USA.; ^4^ Department of Epidemiology and Biostatistics, Michigan State University, East Lansing, Michigan, USA.

**Keywords:** perinatal buprenorphine use, cesarean delivery, medications for opioid use disorder (MOUD) during pregnancy, opioid use disorder (OUD), perioperative pain management, pregnancy

## Abstract

**Background:**

Increasing numbers of pregnant women are being treated with buprenorphine for opioid use disorder (OUD), which can interfere with effectiveness of other opioids used for pain relief, making perioperative guidance for patients requiring cesarean delivery unclear.

**Methods:**

Using a retrospective cohort design, we abstracted 8 years of medical records (2013–2020) from a hospital in rural Michigan. We compared analgesic use (as a proxy for pain) and hospital length of stay (LOS) between groups of women with OUD whose buprenorphine treatment was (1) discontinued before cesarean delivery (discontinuation) versus (2) continued throughout the perioperative period (maintenance). We used *t*-tests and Fisher's Exact tests for comparison of continuous and categorical variables, respectively.

**Results:**

Maternal characteristics reflected the local population (87% non-Hispanic White; 9% American Indian). Of 12,179 mothers giving birth during the study timeframe, 87 met all inclusion criteria (2.4% with diagnosed OUD; 38% of those delivered by cesarean; 76% of those received prenatal buprenorphine treatment). Using the first 2 days of the hospital stay as the standard time window for comparison, there were no differences in perioperative opioid analgesic use (mean ± standard deviation [SD] = 141.6 ± 205.4 vs. 134.0 ± 136.3 morphine milligram equivalents, *p* = 0.89) or LOS (mean ± SD = 2.9 ± 0.9 vs. 3.3 ± 1.0 days, *p* = 0.14) between discontinuation (*n* = 17) versus maintenance (*n* = 70). There was a lower use of acetaminophen in the discontinuation group (mean ± SD = 3,842.6 ± 2,108.1 vs. 4,938.2 ± 2,008.4 mg, *p* = 0.0489).

**Conclusion:**

This study provides empirical evidence supporting continued buprenorphine treatment for women with OUD throughout the perioperative period of a cesarean delivery in a rural setting, although replication with larger sample sizes would provide more confidence in the results.

## Introduction

Overdose deaths involving opioids, including prescription opioids, heroin, and synthetic opioids (like fentanyl), have increased more than sixfold in the US since 1999; by 2020, there were more than 68,000 annual opioid overdose deaths.^1^ These deaths are a marker of the high prevalence of opioid use disorder (OUD) across all population subgroups. Among pregnant women, the US prevalence of OUD more than quadrupled from 1.5 per 1,000 delivery hospitalizations in 1999 to 6.5 per 1,000 delivery hospitalizations in 2014,^2^ and is likely higher now in 2022.

Given the high prevalence of OUD, increasing numbers of pregnant women are being treated with medications for opioid use disorder (MOUD),^1,2^ defined as medication (*i.e.,* buprenorphine, methadone, or naltrexone) shown to sustain recovery and prevent overdose among patients with OUD, including pregnant women.^3,4^ While the Substance Abuse and Mental Health Services Administration (SAMHSA) and the American College of Obstetricians and Gynecologists (ACOG) recommend either buprenorphine or methadone for first line options for pregnant women with OUD, the decision on which MOUD to initiate is often based on provider patient communication and access.^3–5^ Methadone must be administered at SAMHSA-certified treatment programs, and the demands of such daily dosing regimens are often infeasible for pregnant women living in rural areas far from methadone clinics.^6^ Buprenorphine, however, can be prescribed for the treatment of OUD in outpatient settings other than opioid treatment programs, making it often the most accessible MOUD to rural women.^7^

While consistent clinical guidance recommends methadone continuation throughout the perioperative period, buprenorphine continuation postcesarean delivery remains controversial.^8,9^ Because buprenorphine is a partial opioid agonist with high affinity to the mu-opioid receptor, there are concerns that buprenorphine continuation may block the effects of opioids administered for acute pain relief postdelivery, resulting in more opioid consumption.^8–10^ Because clear and consistent perioperative pain management recommendations for women treated with buprenorphine are lacking, our objective was to provide empirical evidence comparing postdelivery analgesic requirements for women with OUD whose buprenorphine treatment was (1) discontinued before cesarean delivery (discontinuation) versus (2) continued throughout the perioperative period (maintenance).

## Methods

We conducted a retrospective cohort study covering 8 years (2013–2020) of patient care for women diagnosed with OUD at a rural medical center serving a large geographic region in Michigan. Length of maternal hospital stay, daily postdelivery morphine milligram equivalents (MME), and daily nonopioid analgesic use were calculated and compared between the groups of patients whose buprenorphine treatment was (1) discontinued before cesarean delivery (discontinuation) versus (2) continued throughout the perioperative period (maintenance). Patient data were abstracted from electronic billing records and transferred to a research database maintained by the study team. To identify the study population, International Classification of Diseases (ICD)-9 and ICD-10 diagnosis codes related to opioid use listed as either a primary or secondary diagnosis were used.

Table 1 lists the diagnoses present in the final study population. The population was then narrowed down to mothers who delivered *via* cesarean as the documented delivery method. We also collected baseline data on women from the hospital admission at the time of delivery/birth, including maternal age, marital status, race, insurance provider, and geographic region.

**Table 1.** International Classification of Diseases (ICD)-9 and ICD-10 Codes Present in Final Study Population

ICD code	ICD code definition
F11.10	OPIOID ABUSE, UNCOMPLICATED
F11.11	OPIOID ABUSE, IN REMISSION
F11.20	OPIOID DEPENDENCE, UNCOMPLICATED
F11.90	OPIOID USE, UNSPECIFIED, UNCOMPLICATED
T40.2X5A	ADVERSE EFFECT OF OTHER OPIOIDS, INITIAL ENCOUNTER
F11.23	OPIOID DEPENDENCE WITH WITHDRAWAL
F11.21	OPIOID DEPENDENCE, IN REMISSION
F11.29	OPIOID DEPENDENCE WITH UNSPECIFIED OPIOID-INDUCED DISORDER
305.50	OPIOID ABUSE-UNSPEC
304.03	OPIOID DEPENDENCE-REMISS
304.01	OPIOID DEPENDENCE-CONTIN
305.53	OPIOID ABUSE-IN REMISS
304.00	OPIOID DEPENDENCE-UNSPEC

ICD, International Classification of Diseases.

Data regarding pain management were extracted from review of the electronic medication administration record and length of stay (LOS) was also collected. All opioid analgesics (butorphanol, fentanyl, hydrocodone, hydromorphone, morphine, oxycodone, and tramadol) were converted to morphine equivalent dose using conversion factors published by the CDC to standardize and compare pain management requirements.^11,12^ This included total dose of opioids received during postoperative hospitalization (excluding buprenorphine). This study was approved by the hospital system Institutional Review Board (15622740-4).

**FIG. 1.**
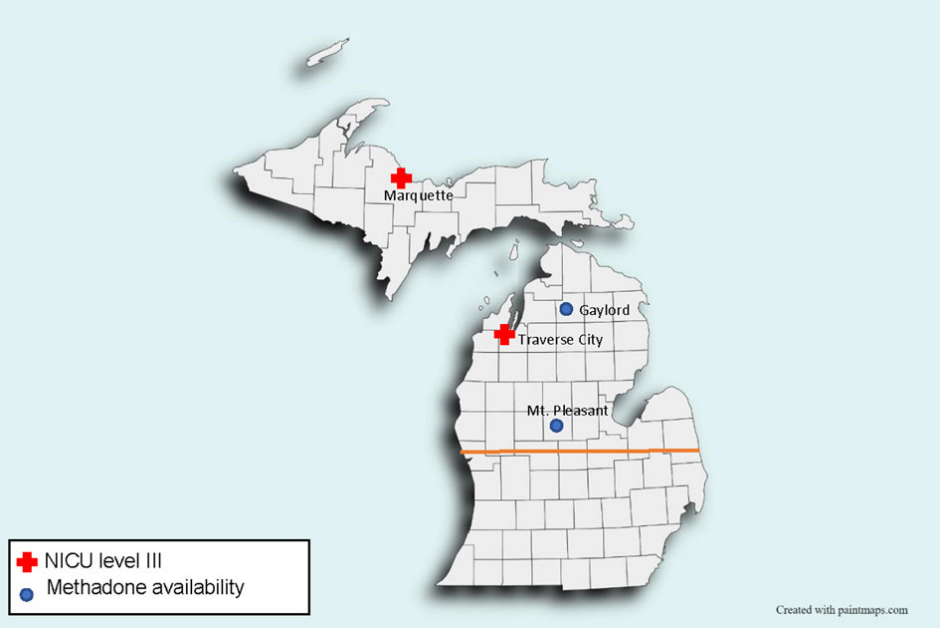
NICU and methadone treatment availability in northern Michigan. Northern Michigan, noted here as the approximate area above the orange line, has only two NICUs (both are level III) and only three methadone treatment centers (two in Gaylord and one in Mt. Pleasant). NICU, neonatal intensive care unit.

Eligible patients included women with OUD who were treated with buprenorphine during pregnancy and delivered *via* cesarean between 2013 and 2020 at a rural medical center. The rural medical center is the only hospital with a neonatal intensive care unit in the region (Fig. 1). As such, patients with high-risk pregnancies travel sometimes long distances to deliver at the hospital, which has a 10,000 square mile catchment area. In this region, methadone treatment is only available at three locations, so most pregnant patients being treated medically for OUD are treated with buprenorphine. Patients who were not being treated with buprenorphine for any reason, including those receiving methadone treatment, were excluded. One patient was excluded because of major chronic medical complications. The decision of whether to continue buprenorphine treatment throughout pregnancy and delivery was made between the patient and the prenatal care provider/obstetric team.

Summary statistics were calculated for all variables to describe patient characteristics. Patients were divided into two groups based on whether their buprenorphine was discontinued before delivery or continued throughout delivery and the postoperative hospital stay. Comparisons between groups used the first 2 days of the hospital stay as the standard time window for comparison and were performed using *t*-tests for continuous variables and Fisher's Exact test for categorical variables due to small cell sizes. For each *t*-test, the assumption of equal variance was checked, and the Satterthwaite method was used when variances were unequal. *t*-Tests completed for all medication variables in this study were deemed to have equal variances, except for MME values, for which we used the Satterthwaite method.

Significance was assessed at *p* < 0.05 for a difference between groups on the primary outcome of MME (a measure of pain management) and the secondary outcomes of maternal length of hospital stay and nonopioid analgesic use. An analysis of power indicated that our study had low power (0.108) to detect a meaningful difference of 22 MME (based on findings from O'Connor et al., 2022) with an alpha of 0.05.^13^ All data analyses were performed using SAS software version 9.4 (SAS Institute, Cary NC).

## Results

**FIG. 2.**
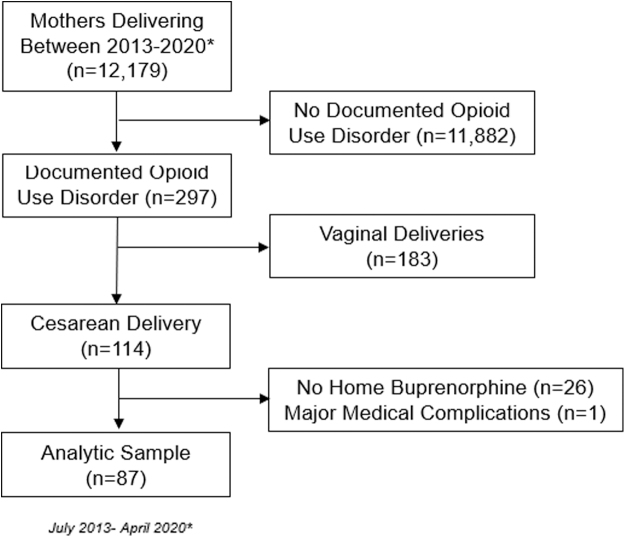
Flow diagram of eligible participants.

During the study time period (2013–2020), 12,179 mothers gave birth at the medical center. Of these, 297 mothers had documented OUD (2.4%), and 114 of those delivered by cesarean. After exclusions were applied (we excluded women who did not receive buprenorphine treatment during pregnancy or received methadone treatment and those with major medical complications), our final analytic sample was 87 (Fig. 2). Maternal characteristics are noted in Table 2 by group, defined as those whose buprenorphine treatment was discontinued during pregnancy before delivery (*n* = 17) versus those whose buprenorphine treatment was continued throughout pregnancy, delivery, and postoperatively after cesarean (*n* = 70).

**Table 2.** Maternal Characteristics Among Women with Opioid Use Disorder Who Delivered *via* Cesarean by Buprenorphine Discontinuation (No BUPE) versus Continuation (BUPE) Before and During Their Delivery Hospitalization: Results from a Rural Health Care System 2013–2020

Characteristics	All ***n*** = 87 ***n*** (%)^a^	No BUPE ***n*** = 17 ***n*** (%)^a^	BUPE ***n*** = 70 ***n*** (%)	***p***
Maternal age (years)				
20–30	51 (58.6)	14 (82.4)	37 (52.9)	
31–41	36 (41.4)	3 (17.7)	33 (47.1)	0.0268
Marital status				
Married/life partner	26 (29.9)	4 (23.5)	22 (31.4)	
Single/divorced/separated	61 (70.1)	13 (76.5)	48 (68.6)	0.5233
Maternal race				
American Indian/Alaska Native	8 (9.2)	0 (0.0)	8 (11.4)	
White	76 (87.4)	16 (94.1)	60 (85.7)	
Not reported	3 (3.5)	1 (5.9)	2 (2.9)	0.2545
Insurance				
Medicare/Medicaid	76 (87.4)	16 (94.1)	60 (85.7)	
Private payer	11 (12.6)	1 (5.9)	10 (14.3)	0.6842
Health care system region				
Grand Traverse County, MI	50 (57.5)	9 (52.9)	41 (58.6)	
Surrounding Service Regions	37 (42.5)	8 (47.1)	29 (41.4)	0.6736

^a^ Column percentages may not add exactly to 100% due to rounding.

OUD, opioid use disorder.

The race of the sample is generally representative of this rural area in the northwest region of Michigan's lower peninsula with 87% self-reporting as White and 9% self-reporting as American Indian/Alaska Native. Most women were unmarried/without a life partner (70%) and had public health insurance (87%). Slightly over half (58%) were from the county where the hospital is located, while 43% lived in the surrounding service region. Maternal age is the only characteristic that varied by buprenorphine continuation with 82% of those whose buprenorphine was discontinued being in the younger age group (20–30 years) versus 53% of those who continued to receive buprenorphine (*p* < 0.05).

Table 3 shows analgesic dose by buprenorphine continuation group using the first 2 days of the hospital stay after cesarean delivery as the standard time window for comparison. The mean MME dose in each group was very similar (141.6 ± 205.4 vs. 134.0 ± 136.3, *p* = 0.8869), but the large standard deviations (SDs) are reflective of the large MME range, reflecting differences in clinical management between patients presumably because of individual patient needs (0–863 MME in the first 2 days and 0–1,160 MME throughout the entire hospital stay). There were no clinically meaningful or statistically significant differences in morphine equivalent dose of opioid analgesics (primary outcome) or LOS (secondary outcome) by buprenorphine continuation.

**Table 3.** Differences in Total Pain Medications Administered for the First 2 Days of the Hospital Stay After Cesarean Delivery to Mothers with Opioid Use Disorder Who Received Buprenorphine During Their Hospital Stay (BUPE) and Mothers Who Did Not Receive Buprenorphine During Their Hospital Stay (No BUPE)

Characteristics	No BUPE ***n*** = 17 Mean (SD)	BUPE ***n*** = 70 Mean (SD)	***p***
LOS (days)	2.9 ± 0.9	3.3 ± 1.0	0.1358
Total MME	141.6 ± 205.4	134.0 ± 136.3	0.8869
Total ketorolac (mg)	130.6 ± 43.7	138.4 ± 42.5	0.4993
Total acetaminophen (mg)	3,842.6 ± 2,108.1	4,938.2 ± 2,008.4	0.0489
Total ibuprofen (mg)	317.6 ± 479.9	205.7 ± 477.9	0.3892
Total BUPE (mg) in hospital	0 ± 0.0	23.3 ± 14.6	<0.0001

BUPE, buprenorphine; LOS, length of stay; MME, morphine milligram equivalents.

For the secondary outcome of nonopioid analgesics, there were no significant differences in dose of ketorolac or ibuprofen by buprenorphine continuation, but acetaminophen dose per day of the hospital stay was slightly higher in the group whose buprenorphine was continued (mean = 4,938.2 ± 2,008.4 vs. 3,842.6 ± 2,108.1 mg; *p* = 0.0489).

## Discussion

Although there are concerns that continuing buprenorphine for treatment of OUD throughout the perioperative period may not allow for adequate pain management after cesarean delivery, we found no differences in pain medication use in mothers who continued buprenorphine use throughout delivery when compared to those who did not. We assumed that this absence of a difference in pain medication use resulted from no differences in pain experienced by the two groups, and that if there were higher levels of pain, it would be reflected in higher opioid use. A measure of pain management (*e.g.,* pain scores) would have been ideal in testing this assumption, but pain scores were not readily availabe patient medical records. This is a major limitation of the study, as similar MME scores would be interpreted quite differently if one group was experiencing significantly more pain (*via* pain scores) than the other group.

Those with OUD have been shown to experience higher pain scores and higher opioid consumption after cesarean delivery compared to those without OUD, so further discerning any effect that buprenorphine continuation throughout delivery may have on pain management and opioid requirements is of clinical importance.^13,14^ Previous research does demonstrate that women maintained on buprenorphine can obtain adequate pain control after vaginal and cesarean delivery with analgesic requirements similar to those required by women maintained on methadone.^9,10,13^

Research on buprenorphine continuation through other surgical operations (*i.e.,* not cesarean delivery) is mixed, with some case reports describing poor postoperative pain control and others finding successful postoperative pain control when buprenorphine was continued.^15^ There remains a need for clear perioperative pain management guidance for individuals with OUD who are taking buprenorphine and undergoing cesarean delivery, and such guidance must take into account both the pharmacodynamic properties of buprenorphine and the patient's desire to maintain stable recovery.

Discontinuing buprenorphine can induce fear and anxiety in pregnant women who are stable in their recovery, and the concern that women on buprenorphine cannot achieve adequate pain control after delivery is the main reason that clinicians discontinue buprenorphine use at delivery.^16^ By demonstrating that women maintained on buprenorphine throughout delivery did not utilize more analgesics than those who discontinued buprenorphine, this study supports the clinical decision not to discontinue buprenorphine use due to pain concerns alone. These results may be especially important to rural physicians whose patients may be more likely to utilize buprenorphine as a MOUD.

During prenatal care, the patient and provider should create a pain management plan together.^17,18^ Throughout this shared patient-provider decision-making process, physicians should also be attuned to how acute pain leads to chronic pain if not treated. Physicians have a responsibility to identify pain management regimes that adequately meet the needs of patients with OUD. Such regiments may include maximizing other pain medications (*e.g.,* ketamine or lidocaine) or consulting with pain specialists.

Because overdose rates are high in the postpartum year, substance use treatment throughout pregnancy is critical.^19,20^ A recent cohort study demonstrated that women with OUD who received MOUD throughout the entire pregnancy had an increased probability of continuing MOUD use for the year after birth.^20^ Thus, buprenorphine continuation throughout delivery may better enable the mother to maintain recovery.

Postdelivery pain management among women with OUD remains understudied. Many research questions on the topic, including ours, are informed by expert clinical insight that identified a gap in recommendations. While such expert opinion should not be undervalued, participatory-based qualitative research to better identify pain management concerns and potential solutions directly from mothers with lived experience with OUD are needed.

Strengths of this study include the use of a retrospective cohort design to provide empirical evidence supporting buprenorphine continuation throughout cesarean delivery in a rural population. Because there is a lack of consistent pain management recommendations for pregnant women on buprenorphine, the use of the retrospective design with chart review allows this important gap in clinical recommendations to be assessed quickly. This retrospective analysis is important because there is a dearth of empirical evidence on this specific topic of buprenorphine maintenance versus discontinuation throughout the peripartum period of a cesarean delivery.

Our sample size and large SDs are not unusual for clinical observations on this topic, for example, Vilkins et al., describe 9 years of clinical data from a large urban medical center with 88 women treated with buprenorphine with a postoperative MME mean (SDs) of 85.1 (73.0).^9^ Our results are somewhat higher and more variable, but they are empirical data and represent a rural population that is often underrepresented in research because larger sample sizes are not possible without increasing the number of years under study. However, the retrospective design over 8 years of clinical data also has limitations. As hospital protocols inevitably change throughout the years, there is the possibility of heterogeneity in prescribing practices over time.

In addition, this study incorporated data that used both ICD-9 and ICD-10 codes, and there may be disruptions in observed rates related to the coding transition. Data from ICD codes are also subject to coding errors, unmeasured confounding, misclassification bias, missing data, and changing participant eligibility over time. We also did not have an assessment of opiate withdrawal or any other postdelivery indicator of wellbeing (*e.g.,* breastfeeding success, depression scores) that would demonstrate potential benefits of buprenorphine continuation from a risk-reduction standpoint. Similarly, we were not able to follow women postdischarge to assess buprenorphine continuation or any associated relapse rates. By using analgesic use as a proxy for pain management, we made assumptions that the mothers' pain experiences were similar by buprenorphine continuation status, but future studies should consider pain scores where available or other more direct assessments of pain.

As with any observational study, the use of a nonrandomized design permits the possibility of confounding, and the presented results were unadjusted partially due to our small sample size that made stratification unfeasible. The buprenorphine continuation and buprenorphine discontinuation groups were comparable in most important sociodemographic variables, but the age distribution did differ significantly by buprenorphine continuation status, possibly allowing for residual confounding. Larger prospective studies should seek to replicate this study with more adequate confounder adjustment. Finally, we did not consider differences in maintenance buprenorphine doses that patients were receiving before their cesarean delivery.

## Conclusion

In this retrospective cohort covering 8 years of patient care in a rural population, no differences in opioid analgesic use—used here as a proxy for pain—were found whether buprenorphine was stopped before delivery (*n* = 17) or continued throughout the perioperative period (*n* = 70). Existing clinical guidance for buprenorphine continuation throughout the perioperative period is based on the theoretical pharmacodynamic properties of opioids, but this study provides empirical evidence to support the use of continued buprenorphine treatment for women with OUD throughout the perioperative period of a cesarean delivery and should help inform clinical guidance on postdelivery pain management among women with OUD.

## Abbreviations Used

ACOG: American College of Obstetricians and Gynecologists
BUPE: buprenorphine
ICD: International Classification of Diseases
LOS: length of stay
MAR: medication administration record
MME: morphine milligram equivalents
MOUD: medications for opioid use disorder
NICU: neonatal intensive care unit
OUD: opioid use disorder
SAMHSA: Substance Abuse and Mental Health Services Administration
SDs: standard deviations
